# Relationship between salivary stress biomarker levels and cigarette smoking in healthy young adults: an exploratory analysis

**DOI:** 10.1186/s12971-016-0085-8

**Published:** 2016-06-06

**Authors:** Nao Suzuki, Kosuke Nakanishi, Masahiro Yoneda, Takao Hirofuji, Takashi Hanioka

**Affiliations:** Department of Preventive and Public Health Dentistry, Fukuoka Dental College, 2-15-1 Tamura, Sawara-ku, Fukuoka, 814-0193 Japan; Department of General Dentistry, Fukuoka Dental College, 2-15-1 Tamura, Sawara-ku, Fukuoka, 814-0193 Japan

**Keywords:** Cigarette smoking, Interleukin-1β, Profile of mood states, Secretory immunoglobulin A, Salivary stress markers, Tumor necrosis factor-α

## Abstract

**Background:**

This study investigated the relationships among salivary stress biomarkers, cigarette smoking, and mood states.

**Methods:**

The study population comprised 49 healthy sixth-year dental students at Fukuoka Dental College (39 men, 10 women; age, 23–31 years). Lifetime exposure to smoking was calculated using the Brinkman index (BI). Resting saliva samples were collected, and concentrations of cortisol, secretory immunoglobulin A (SIgA), interleukin (IL)-1β, interleukin-6, and tumor necrosis factor (TNF)-α were determined. Mood states (tension-anxiety, depression-dejection, anger-hostility, fatigue, confusion, and vigor) over the previous week were assessed using the Profile of Mood States - Brief Japanese Version.

**Results:**

Salivary IL-1β levels were significantly higher in smokers than non-smokers (*P* = 0.044), regardless of the BI or mood state. A significant positive correlation was evident between the TNF-α level and the BI (*P* = 0.036), and the SIgA level was positively correlated with the BI (*P* = 0.067) but did not reach statistical significance. In terms of mood states, higher fatigue scores and lower vigor scores were observed in smokers. The TNF-α level and vigor score were negatively correlated (*r* = –0.229, *P* = 0.135), but the correlation did not reach statistical significance. However, the SIgA level and fatigue score were significantly positively correlated (*r* = 0.410, *P* = 0.005).

**Conclusions:**

The TNF-α and SIgA levels were both positively correlated with the BI. Furthermore, the TNF-α level was negatively correlated with the vigor score, whereas the SIgA level was positively correlated with the fatigue score. Thus, salivary levels of TNF-α and SIgA may be used as biomarkers of mood states in healthy young smokers.

## Background

Smokers generally indicate that stress relief and relaxation are their main reasons for smoking [1, 2]. Previous experimental studies have found that cigarette craving is increased after acute stress exposure in smokers [3, 4]. Furthermore, an increased rate of smoking is reported after large-scale disasters, such as terrorist attacks, hurricanes, and fires [5–7]. On the other hand, chronic exposure to nicotine is thought to increase subjective stress levels and exacerbate a depressed mood by inducing changes in neurotransmitter systems and neural pathways implicated in mood regulation. Associations between smoking and negative moods, symptoms of terminal insomnia, and psychiatric disorders have been reported in clinical and epidemiologic studies [8–10].

The hypothalamic-pituitary-adrenal (HPA) and sympathetic-adrenal-medullary (SAM) axes are the two main stress response pathways in the human body. In addition to these axes, a link between the immune system and stress has been reported recently [11]. Bidirectional communication between the HPA axis and immune system plays a key role in the response to chronic and repeated stressors. Cortisol, which is an effector of the HPA axis, increases after nicotine administration and decreases in response to acute tobacco abstinence [12, 13]. In the context of chronic stress, salivary cortisol levels are higher in depressed individuals, compared to non-depressed individuals [14]. The effect of smoking-associated chronic stress on salivary cortisol production is unclear. The glycoprotein secretory immunoglobulin A (SIgA) participates in the acquired immune response. Under acute stress, the SIgA levels in saliva change in association with cortisol levels [15]. By contrast, other research suggests an inverse association between SIgA and cortisol following psychological stress [16]. Salivary SIgA levels are generally recognized to decrease in response to chronic stress, but increase in response to acute stress. There are various reports on the relationship between cigarette smoking and salivary SIgA. Some studies have reported lower levels of salivary SIgA in smokers, compared to non-smokers [17, 18]. However, another study reported increased levels of SIgA in the saliva of immunocompetent smokers [19]. Proinflammatory cytokines, including interleukin (IL)-1β, interleukin (IL)-6, and tumor necrosis factor (TNF)-α, which increase during early inflammation, are believed to activate the HPA axis [20]. The effects of cigarette smoking on the levels of these cytokines in gingival crevicular fluid (GCF) have been researched extensively in relation to periodontitis [21]. Some reports indicate that high levels of these cytokines are observed in the GCF of smokers [22, 23]. Conversely, the GCF level of IL-1β levels at deep bleeding sites were reported to be lower in smokers than in non-smokers [24]. In another study, the TNF-α levels in the GCF were found to be higher in smokers than in non-smokers, although smoking was not associated with differing levels of IL-6 in the GCF [25]. There are few reports of the use of salivary cytokines to evaluate the effects of smoking on the immune response in healthy participants.

In this study, we investigated the relationship between the levels of five salivary stress biomarkers (cortisol, SIgA, IL-1β, IL-6, and TNF-α) and cigarette smoking in healthy young adults. Furthermore, the relationship between salivary biomarker levels and mood states related to cigarette smoking was explored.

## Methods

### Study population

The study population consisted of 49 subjects (39 men, 10 women; mean age, 25.6 ± 2.1 years; range, 23–31 years) who were healthy sixth-year dental students at Fukuoka Dental College. Of the 51 students assigned to participate in the study, 1 student refused to participate and another student did not provide sufficient resting saliva for analysis. Therefore, 49 students participated in the study. Approval of this study was obtained from the Ethics Committee for Clinical Research at Fukuoka Dental College and Fukuoka College of Health Sciences (Approval No. 249). All participants understood the nature of the research project, and provided written informed consent to participate in this study.

Samples were collected in June 2014. No problems were found for any participant in dental and health checkups performed in April 2014. No participant had taken antibiotics for at least the past 3 months. The smoking status of the participants was obtained by a self-completed questionnaire. Smoking status was defined in the questionnaires as “smokers”, individuals who had smoked ≥100 cigarettes in total after starting smoking and “non-smokers”, individuals who had never smoked or smoked <100 cigarettes in total after starting smoking [26]. Lifetime exposure to smoking was calculated using the Brinkman index (BI), which is defined as (number of cigarettes per day) × (number of years for which a person smoked) [27].

### Collection of saliva, and determination of cortisol, SIgA, IL-1β, IL-6, and TNF-α concentrations

Participants were asked to collect 3 mL of resting whole saliva in a disposable plastic tube at 3:30 pm at least 2.5 h after eating, smoking, or brushing their teeth. The saliva samples were stored at –30 °C until biomarker concentrations were measured.

The concentrations of salivary stress biomarkers were determined using enzyme-linked immunosorbent assays (ELISAs). The cortisol and SIgA concentrations were quantified by competitive immunoassays using the Cortisol ELISA kit and Secretory Immunoglobulin A ELISA kit, respectively (Salimetrics LLC, State College, PA, USA). IL-1β, IL-6, and TNF-α concentrations were quantified by sandwich ELISA, using the Human IL-1β ELISA, Human IL-6 ELISA, and Human TNF-α ELISA kits, respectively (Diaclone, Besançon Cedex, France).

### Mood assessment

Mood states over the previous week were assessed using the Profile of Mood States (POMS) - Brief Japanese Version (Kaneko Publishing, Tokyo, Japan). The POMS short form evaluates moods on six subscales (Tension-Anxiety, Depression-Dejection, Anger-Hostility, Fatigue, Confusion, and Vigor) in a short time [28]. The instrument contains 30 items that are rated on a 5-point scale from 0 (not at all) to 4 (extremely). Elementary scores were converted to T scores according to the sex- and age-specific table that was provided by the manufacturer. Each T score was calculated as follows: 50 + 10 × (elementary score – average)/standard deviation.

### Statistical analysis

The *t*-test and χ^2^ test were used to compare demographic and mood state data between smokers and non-smokers. Linear regression analysis, adjusted for age, was used to compare the concentrations of salivary stress biomarkers between smokers and non-smokers. Partial correlation coefficients were calculated to evaluate the relationships among salivary stress biomarker levels, BI, and age and among salivary stress biomarker levels, mood state, and age. All statistical analyses were performed using SPSS software (version 22.0; SPSS Japan, Tokyo, Japan). A *P* value of <0.05 was considered statistically significant.

## Results

There were 18 smokers and 31 non-smokers. The smoker group comprised 17 current smokers and one ex-smoker who had stopped smoking 1 year prior. The average age of the smokers (26.8 ± 2.4 years [± SD]) was significantly greater than that of the non-smokers (25.0 ± 1.6 years [± SD]) (*P* = 0.005). The proportions of women were 11.1 % (2/18) in the smoker group and 25.8 % (8/31) in the non-smoker group, with no significant difference between the two groups (*P* = 0.219).

**Fig. 1:**
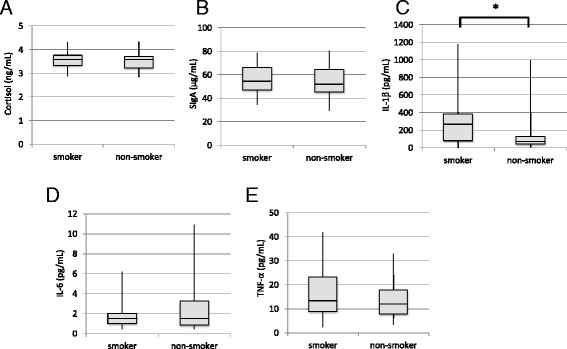
Comparison of the levels of salivary stress biomarkers between smokers and non-smokers: cortisol (**a**), SIgA (**b**), IL-1β (**c**), IL-6 (**d**), and TNF-α (**e**). *P < 0.05 by linear regression analysis after adjusting for age

Figure 1 compares salivary stress biomarkers between the smokers and non-smokers after adjusting for age. The concentrations (median [IQR]) of IL-1β were significantly (*P* = 0.044) higher in the smokers than non-smokers (271.3 [81.0–385.2] vs. 74.2 [44.6–131.6] pg/mL, respectively). Although the concentrations of SIgA (54.4 [47.1–66.3] vs. 52.7 [45.3–64.7] μg/mL) and TNF-α (13.7 [8.90–23.3] vs. 12.5 [7.82–17.9] pg/mL) were slightly higher in the smokers compared to the non-smokers, the differences were not significant. The cortisol and IL-6 concentrations in saliva did not differ between the two groups. The range of IL-6 levels was greater in the non-smokers than in the smokers.

**Fig. 2:**
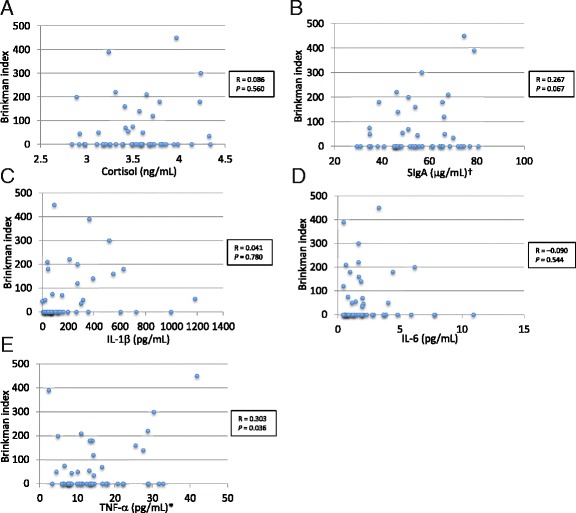
Correlation between salivary stress biomarker levels and the Brinkman index after adjusting for age: cortisol (**a**), SIgA (**b**), IL-1β (**c**), IL-6 (**d**), and TNF-α (**e**). R: partial correlation coefficient. The TNF-α level and the Brinkman index were positively correlated (*P* = 0.036). The SIgA level was weakly positively correlated with the Brinkman index, but statistical significance was not attained (*P* = 0.067)

Subsequently, the relationships between the concentrations of salivary stress biomarkers and the BI were analyzed after adjusting for age. The median [IQR] BI in the smokers was 150 [58.8–207.5]. Figure 2 summarizes the correlations between salivary stress biomarkers and the BI in all participants, with the BI of non-smokers set to 0. TNF-α concentrations had a significantly positive correlation with BI values (*r* = 0.303, *P* = 0.036). There was a weak positive correlation trend between the concentration of SIgA concentrations and the BI value (*r* = 0.267, *P* = 0.067), but statistical significance was not observed. The other salivary biomarkers showed no correlation with BI values.

Analysis of the mood states obtained from the POMS showed higher fatigue scores (*P* = 0.047) and lower vigor scores (*P* = 0.017) in the smokers compared to the non-smokers (Table 1). In addition, the scores for depression-dejection, anger-hostility, and confusion were higher in the smokers compared to the non-smokers, but without statistical significance. The correlations among salivary biomarkers, the mood states of vigor and fatigue related to smoking, and age were explored (Table 2). TNF-α levels showed a weak non-significant negative correlation with vigor (*r* = –0.229, *P* = 0.135). SIgA levels showed a significant positive correlation with fatigue (*r* = 0.410, *P* = 0.005).

**Table 1:** Mood states, as evaluated using the POMS (T scores, mean ± SD)

POMS scale	Non-smokers (*n* = 31)	Smokers (*n* = 18)
Tension-Anxiety	54.2 ± 12.4	52.3 ± 12.3
Depression-Dejection	51.2 ± 11.1	57.0 ± 11.9
Anger-Hostility	49.1 ± 9.8	51.0 ± 11.7
Vigor*	46.8 ± 12.5	39.9 ± 6.5
Fatigue*	54.2 ± 14.0	61.9 ± 11.7
Confusion	57.2 ± 13.1	59.7 ± 10.5

*POMS* Profile of Mood States

*P < 0.05 between smokers and non-smokers according to the *t-*test

**Table 2:** Partial correlation coefficients between salivary stress biomarkers, mood states of vigor and fatigue, and age

Salivary stress biomarker	Vigor	Fatigue
Cortisol	-0.188	0.044
SIgA	-0.175	0.410*
IL-1β	-0.176	-0.030
IL-6	0.079	-0.139
TNF-α	-0.229^a^	0.020

*P < 0.05

^a^A weak negative correlation was evident between the TNF-α level and vigor, but statistical significance was not attained (*P* = 0.135)

## Discussion

Salivary cytokines are produced during periodontal inflammation and tissue destruction. Smoking also increases cytokine levels in the saliva and gingival crevicular fluid, accelerates inflammation, and destroys periodontal tissue. Therefore, many studies have focused on the effect of smoking on cytokines in periodontitis, and adult participants, including middle-aged and older people, have generally been targeted. In our current work, we studied periodontally healthy young adults to avoid the effects of the periodontal conditions on the saliva biomarker concentrations. The primary findings of this study were that salivary IL-1β is associated with active smoking, independent of the amount smoked, and that salivary TNF-α levels positively correlate with the amount smoked.

The median IL-1β concentration was 3.6 times higher in the smokers (271 pg/mL) than in the non-smokers (74 pg/mL) in this study. A study on ≥30-year-old participants with periodontitis also reported that the mean IL-1β concentration was higher in smokers (706 pg/mL) than in non-smokers (612 pg/mL) [29]. Also, in the periodontally healthy control group of the cited study, the mean IL-1β concentration was slightly higher in smokers (479 pg/mL) than in non-smokers (464 pg/mL), but the difference was not significant [29]. Overall, there are few data on the effect of smoking on salivary IL-1β levels in periodontally healthy populations. The effects of smoking on salivary IL-1β production are diverse across the whole population. A cross-sectional study in adults (20–89 years) reported that IL-1β levels were significantly higher in participants with periodontitis (144 pg/mL) compared to healthy participants (61 pg/mL), but there was no difference in salivary IL-1β concentrations between smokers (52 pg/mL) and non-smokers (78 pg/mL) [30]. An analysis of salivary cytokines in workers age 18–62 years found that the mean salivary IL-1β level was significantly higher in passive smokers (190 pg/mL) than in non-smokers (164 pg/mL), but no significant difference was found between passive and active smokers (167 pg/mL) [31]. Furthermore, levels of other biomarkers, such as prostaglandin E2, matrix metalloproteinase-9, lactoferrin, albumin, and aspartate aminotransferase were significantly lower in active smokers [31]. Based on these data, smoking may have the potential to suppress the host-defense system and promote periodontal disease progression. The effects of smoking on salivary cytokines likely vary widely with age, host-defense system characteristics, and the progression of periodontitis. Aging is characterized by qualitative and quantitative changes in the immune system (i.e., increased levels of pro-inflammatory cytokines and reduced levels of anti-inflammatory cytokines [32]). Therefore, the age of the study participants may have affected the biomarker levels. The age range of the participants was narrow (23–31 years); therefore, the observed effect of smoking on salivary IL-1β levels is likely to be real, although the age of smokers was significantly higher than that of non-smokers.

In this study, salivary TNF-α concentrations were positively correlated with the amount smoked. Our result is consistent with studies that examined the TNF-α levels in serum and in exhaled breath condensate [33, 34]. Analysis of exhaled breath condensate TNF-α concentrations revealed that smokers had higher TNF-α concentrations, compared to non-smokers [33]. Petrescu et al. [34] reported that the serum TNF-α levels were significantly higher in a group of smokers compared to a group of nonsmokers. They also noticed increased TNF-α concentrations in the serum of smokers that smoked more than one pack per day compared with those who smoked less than one pack per day. The high concentration of TNF-α in smokers is understood to reflect smoke-induced lung injuries and is considered a useful biomarker for the identification of heavy smokers with a high risk of developing smoke-induced pulmonary diseases.

TNF-α concentrations also correlated weakly in a negative manner with vigor scores, which were lower in smokers than in non-smokers. Proinflammatory cytokines (TNF-α, IL-1β, and IL-6) stimulate the HPA axis, promoting the secretion of corticotropin releasing factor, which may be one mechanism by which inflammation induces depressive symptoms [35]. In addition, TNF-α is believed to trigger depressive symptoms through the activation of neuronal serotonin transporters and the stimulation of indoleamine 2,3-dioxygenase activity, causing tryptophan depletion. [36]. Cavadini et al. [37] reported that increased TNF-α and IL-1β levels impaired clock gene functions and caused sleep disturbances and fatigue in an in vivo assay using mice. A clinical study that evaluated the effect of forest bathing found an inverse correlation between TNF-α levels and vigor scores [38]. The high salivary TNF-α levels in the smokers observed in this study could affect mental health.

Chronic stress suppresses salivary SIgA in general. Contrary to expectations, salivary SIgA concentrations showed a weak positive correlation with the amount of smoking and a significant positive correlation with fatigue scores. Norhagen and Engström [19] reported increased SIgA levels in the saliva of immunocompetent smokers, and suggested that this is indicative of an attempt to protect the oral mucosa. Our study participants were physically healthy, so our result might be related to immune protection. Conversely, it has been reported that acute psychological stress caused by a mock job interview increased salivary SIgA levels in undergraduate students [39]. Although a mental load test was not performed in the current study, just before implementation, the participants were informed of the requirement to collect saliva and complete the POMS and smoking-habit questionnaires. Those requirements might have resulted in acute stress for smokers.

Our study had some design limitations. First, the average age of smokers was significantly higher than that of nonsmokers, even though the age range of the study participants was narrow. Therefore, the data in the statistical analyses were adjusted for age. Second, few study subjects were women. There were no differences in salivary biomarker levels between men and women (data not shown). A previous study reported that the SIgA concentration in young children was the same in males and females [40]. However, another study recently reported that SIgA levels in young healthy adults were lower in females than males [41]. The effects of sex on the statistical power and the generalizability of the study results must be interpreted with caution. Finally, saliva collection was performed at 15:30 and at least 2.5 h after eating, smoking, or teeth brushing. Salivary biomarkers have distinctive circadian [42]. If a longitudinal study of salivary biomarkers is conducted, it is important that saliva collection be done at the same time and with the same conditions.

## Conclusions

This study examined physically and mentally healthy young adults. IL-1β and TNF-α levels in saliva were associated with smoking, and analysis of the mood states obtained from the POMS showed higher fatigue scores and lower vigor scores in the smokers than the non-smokers. In addition, a trend toward a negative relationship was observed between TNF-α levels in saliva and vigor scores. The salivary SIgA concentration was weakly positively correlated with the extent of smoking, but was significantly positively correlated with the fatigue score. Salivary levels of TNF-α and SIgA may be useful biomarkers of mood states in healthy young smokers.
